# Exploring Challenges to COVID-19 Vaccination in the Darfur Region of Sudan

**DOI:** 10.4269/ajtmh.21-0782

**Published:** 2021-11-10

**Authors:** Alanood Elnaeem Mohamed, Yasir Ahmed Mohammed Elhadi, Nora Alnaeem Mohammed, Aniekan Ekpenyong, Don Eliseo Lucero-Prisno

**Affiliations:** ^1^ Faculty of Pharmacy, University of Science and Technology, Khartoum, Sudan;; ^2^ Department of Health Administrations and Behavioral Sciences, High Institute of Public Health, Alexandria University, Egypt;; ^3^ Department of Public Health, Medical Research Office, Sudanese Medical Research Association, Khartoum, Sudan;; ^4^ Faculty of Medicine, University of Science and Technology, Khartoum, Sudan;; ^5^ Global Health Policy Units, University of Edinburgh, Edinburgh, United Kingdom;; ^6^ Department of Global Health and Development, London School of Hygiene and Tropical Medicine, London, United Kingdom

## Abstract

The current COVID-19 pandemic has affected the ability of health systems to provide essential services globally. The Darfur region, located in the western part of Sudan, has been largely devastated by the war that began in 2003 and has been drawing considerable attention from the international community. The war, which erupted as a result of environmental, political, and economic factors, has led to tragic outcomes. Collapsing health-care infrastructures, health workforce shortages, lack of storage facilities for medicines and medical products, and inadequate access to health services are some of the effects of the war. After Sudan received the AstraZeneca COVID-19 vaccine through the COVID-19 Vaccines Global Access facility, significant challenges have been implicated in the delivery, storage, and use of the vaccine in the Darfur region. Lack of vaccine storage and transportation facilities, vaccination hesitancy, inequity in the distribution to health facilities, and shortage of health-care professionals resulting from insecurity and instability have added an extra layer of burden on local authorities and their ability to manage COVID-19 vaccinations in the region adequately. Addressing the impact of COVID-19 requires an effectively managed vaccination program. In the face of current challenges in Darfur, ensuring a fully vaccinated population might remain far-fetched and improbable if meaningful efforts are not put in place by all stakeholders and actors to address some of the challenges identified.

## INTRODUCTION

**Figure 1.**
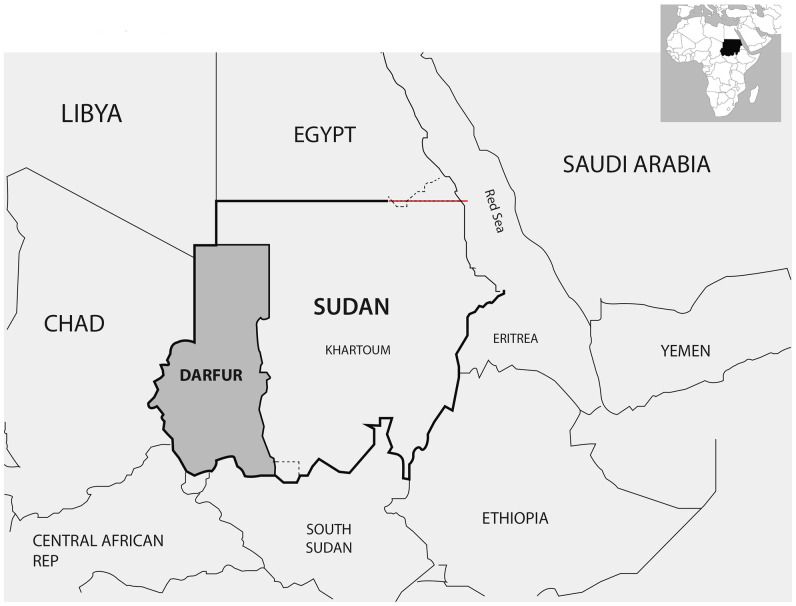
Darfur region in Sudan. This figure appears in color at www.ajtmh.org.

The Darfur region (northern, southern, middle, western, and eastern Darfur states) represents five of the total 17 Sudanese states located in the western part of Sudan (Figure 1). It incorporates a population of roughly 8.2 million from 80 tribes and ethnic groups (2010 estimate). Tribal and ethnic disputes have been a hallmark of the Darfur region long before the start of the devastating civil war in 2003. Mass violence, genocide, and human rights violations have occurred in the region, causing up to 300,000 deaths and 2.5 million displacements according to the United Nations. Widespread destruction has certainly cast a shadow on the health-care services in the region.^1^ The current COVID-19 pandemic is a public health concern on a worldwide scale that has affected the ability of health systems to provide essential services globally.^2^

## COVID-19 VACCINATION PROGRAM IN SUDAN

Sudan received the AstraZeneca COVID-19 vaccine through the COVID-19 Vaccines Global Access (COVAX) facility. More than 1 million doses of the vaccine were supplied, with an immunization supply chain that consisted of the national store, 18 state stores (including Darfur states), 183 local stores, and 2,421 service points. In the Darfur region, the immunization supply chain consists of five stores with adequate capacity and 485 service points. To cover 8.5 million people representing 20% of its population, Sudan requested 17 million doses of the COVID-19 vaccine. In March 2021, more than 800,000 doses for the Sudanese population arrived in two shipments. The doses were prioritized for health-care workers at risk of direct contact with COVID-19 patients, health-care workers 45 years and older, and people 45 years and older with a medical condition and living in areas with high transmission or anticipated high transmission.^3^ To achieve a successful immunization program, various elements need to be in place, including a robust cold-chain system, transport and delivery of vaccines, stock maintenance, training of health workers, advocacy, monitoring, and evaluation.^4^

## CHALLENGES FACING DAFUR’S COVID-19 VACCINATION PROGRAM

Darfur is considered one of the most challenging areas to achieve vaccination because it faces multiple crises. Darfur’s civil conflict has undoubtedly aggravated an immense infrastructure deficit, notably within the transport, water, and power sectors. Significant geographic isolation, poor access to education and health facilities, weak governance, weakening of human and institutional capabilities, low levels of economic activity, and restricted employment opportunities are examples of these deficits.^5^

The conflict has multiplied the demand for health-care services and also decreased the number of health facilities through conflicts among rebels and other violence. From 2002 to 2010, the number of primary health-care centers decreased by 26%, from 82 to 61; clinics by 29%, from 111 to 79; and basic health units by 64%, from 203 to 130. Moreover, there is an absence of technical equipment in public health facilities and in most non-public health facilities.^6^ Although international non-governmental organizations (NGOs) have been providing 70% of health services to the population of Darfur by contributing 52.9% of the health budget and 1,390 health personnel,^7^ the government in the Darfur region faces difficulties in providing curative health services to the population. The budget allocation for health services of overall state expenditure declined steadily from 7.6% in 2000 to 5.6% in 2009. In contrast, the budgetary allocation for security has grown drastically from 23.7% in 2000 to 38.2% in 2009.^8^ With this scenario, and with a large number of internally displaced people and refugees already hosted, the spread of COVID-19 has posed a significant threat to the health-care system.^9^

The fragile health system has increased the burden of communicable diseases such as tuberculosis (TB), and other diseases of concern such as cholera, Rift Valley fever, and hepatitis.^10^ The Ministry National Program for Combating Tuberculosis confirmed a wide spread of the disease. In 2019, 20,164 cases were reported. Considering that the rate of detection was 60% to 64%, nearly 36% to 40% of the people suffering from TB have not been diagnosed. Because of issues with not being able to access health institutions and the lack of monitoring, the complete outcome rates and risk of TB on the Darfur population are uncertain.^11^ Because the TB situation has been this terrible as a result of armed conflicts and other challenges, it is most likely that the pandemic and vaccination might follow a similar path.

### Vaccine storage and transportation.

The cold-chain system in Darfur is not optimal for storage of vaccines and might affect the quality of the vaccines being stored. Also, the electric and power supply is inadequate and does not meet needs and demands.^5^ In addition, scarcity of petroleum products and solar power affects the storage of the Oxford–AstraZeneca vaccine, requiring temperatures of 2 to 8°C for its storage. The transportation of COVID-19 vaccines is not incorporated in the Expanded Program on Immunization–Immunization Supply Chain. Furthermore, there are no clear plans and budget allocated for vaccine distribution at lower levels of the health-care system. In addition, a lack of vehicles with refrigerators, an inadequate distribution fleet to match the expansion in Expanded Program on Immunization services at the state level primarily, and poor road infrastructure make it challenging to effect adequate distribution. The storage facilities lack the essentials for proper storage of the vaccine. Despite requesting more doses of the vaccine from COVAX, the expected increase in capacity required to store and distribute the vaccine has not been met by an increase of resources to mobilize and procure new equipment for proper storage of the vaccines.^12^

### Health workforce shortage.

The number of health-care workers is decreasing remarkably throughout the region as a result of long-term insecurity and lack of funding. This is another challenge in the vaccination process. Darfur’s inhabitants face further worsening of already limited health care—only 0.4 health providers per 1,000 population compared with the WHO benchmark of 2.3 providers per 1,000 population. Also, the lack of funding and adequate remuneration is causing a marked decrease in health facilities as NGOs are being forced to withdraw their vital services. Most of the primary health-care facilities lack the minimum required medical equipment and a robust infrastructure.^13^ Regardless of the presence of good vaccine storage facilities and transport capacity, the lack of a competent, trained health workforce to administer the vaccine to the population hinders progress in this regard.^14^ Despite the efforts of international agencies to support protracted, complex, and overlapping humanitarian challenges in Darfur, a remaining gap to be addressed is how to attract and retain qualified health personnel to work in this region.^15^

### Inequity in the distribution of health facilities.

Another serious issue in Darfur is the inequity in the geographic distribution of health facilities. Several rural communities are underserved by the health system and lack access to basic health services.^13^ Rehabilitating existing health facilities, and increasing the number of trained health professionals with effective retention and remuneration strategies are key priorities. Furthermore USD 105 million are required to sustain health and nutrition coverage over the ensuing 15 years.^13^ In the meantime, with no lasting peace in the foreseeable future and the economic meltdown, Darfur inhabitants need to use existing resources and hope for stronger international support.

### Vaccination hesitancy.

Hesitancy toward vaccination against COVID-19 is another challenge that needs to be addressed. In Sudan, the spread of misinformation regarding the COVID-19 pandemic has manifested in vaccination hesitancy among health-care workers and the public.^16^ The rates of COVID-19 vaccination hesitancy are high throughout Sudan. A recent study reported 43.8% prevalence of vaccination hesitancy in Sudan among health workers. The study concluded that the unreliability of COVID-19 vaccine clinical trials and worry about the vaccine’s side effects were reported as perceived reasons behind COVID-19 vaccination hesitancy in the region.^17^ Distrust in government by the public remains an outstanding barrier to the efforts of government and health authorities in promoting COVID-19 vaccination and building vaccination confidence. This could be related to the low level of education among the people of this region. Evidently, higher education and a history of comorbidities leads to lower hesitancy rates.^18^ Recent events involving the Oxford–AstraZeneca COVID-19 vaccine likely contributed to vaccination hesitancy, as the use of this vaccine was suspended in more than 20 countries because of potential vaccine-induced prothrombotic immune thrombocytopenia.^19^

Risk communication and community engagement (RCCE) is an important strategy for building resilient local health systems, ensuring equity for all, and responding to the needs of the population, especially underserved groups as internally displaced persons, returnees, and rural communities.^20^ The establishment of an RCCE system in Darfur would be beneficial in reducing the rate of transmissibility and spread of the virus, especially in highly vulnerable communities. According to the Humanitarian Country Team/United Nations Country Team COVID-19 Country Preparedness and Response Plan, which was set up to assist the government of Sudan during the current pandemic, one of the main objectives is to reinforce the protocols of the RCCE system. Some of the major elements of this scheme are the ability to engage with the local communities directly through media, local NGOs, schools, and local governments; to promote effective collaboration; and to use methods to enforce protocols for COVID-19 prevention.^21^

A real-life example that signifies the impact of the community engagement schemes was conducted in June and July 2020 in the Darfur region, where, surprisingly, the data revealed that the African Union–United Nations Hybrid Operation in Darfur Communication and Public Information Section Month-long Street Publicity Awareness Campaign resulted in beneficial effects in increasing awareness about the disease, including its transmission and prevention in more than 1 million residents from the five states of Darfur. This awareness campaign was able to broadcast data about COVID-19 and was successfully implemented in towns, neighborhoods, markets, and 10 internally displaced population camps across Darfur.^22^

## RECOMMENDED CONTEXTUAL SOLUTIONS

Although a major step has been achieved with the successful dissemination of the COVID-19 vaccine to the region of Darfur, multiple hindrances still pose problems with regard to the effective implementation of the vaccine dissemination process. To ensure improvement in the use of vaccines, there is a need for support schemes from the government. Furthermore, it is essential that monetary support be made available to support the dissemination process in the Darfur region. International NGOs working in the region alongside the government are key to achieving this objective.

There is a need to focus efforts on the establishment of a classification system to enable categorization of residents in Darfur. Doing this enables the identification of the large number of displaced people and refugees who mostly are not registered and do not have legal documents, and hence might fall through the cracks during vaccination. Such a system may also assist in overcoming inequities in health-care distribution in Darfur by identifying the general distribution pattern, making it possible to extend vaccination coverage to the greatest number of residents.

Furthermore, a significant proportion of the budget allocated to the health system in Darfur needs to be reserved to facilitate effective storage and transport of the vaccines. In current circumstances, it may seem impossible to make huge repairs in the region’s devastated infrastructure. However, feasible solutions exist, such as construction of temporary refrigeration systems to assist in preserving the vaccines’ efficacy and quality. In addition, it is important to establish strong security plans to prevent unlawful access to the vaccines at storage and administration centers.

Regarding the shortage of health-care workers in the region, NGOs could play an essential role in recruiting and training volunteers and health workers from across Sudan. This should be done alongside addressing the challenge of vaccination hesitancy. As shown previously in multiple surveys, hesitancy toward procuring the COVID-19 vaccine is a global threat in several countries. Therefore, it is necessary that appropriate educational campaigns to counter vaccination hesitancy be rolled out throughout the region to increase confidence in vaccine efficacy.

## CONCLUSION

It is clear that the COVID-19 vaccination program in Darfur is being limited by numerous challenges, making it difficult to coordinate the vaccination process effectively. Some of the challenges identified include a lack of infrastructure for the storage and transportation of vaccines, a health workforce shortage, inequity in the distribution of health facilities, and vaccination hesitancy. Hence, there an urgent need to design solutions that can address the issues identified, and to do so in collaboration with all relevant stakeholders, including local health authorities, national authorities, and the humanitarian sector.
